# Five-years experiences of the Revised National Tuberculosis Control Programme in northern part of Kolkata, India

**DOI:** 10.4103/0970-2113.56343

**Published:** 2009

**Authors:** Sudipta Pandit, Atin Dey, Arunabha Datta Chaudhuri, Mita Saha, Amitava Sengupta, Sushmita Kundu, Sourin Bhuniya, Shib Singh

**Affiliations:** *Department of Chest Medicine, R.G. Kar Medical College and Hospital, Khudiram Bose Sarani, Kolkata-4, India*; 1 *Department of Community Medicine, R.G.Kar Medical College and Hospital, Kolkata, India*; 2 *Department of Chest Medicine, North Bengal Medical College and Hospital, Darjeeling, India*; 3 *Department of Chest Medicine, Midnapore Medical College and Hospital, Midnapore, India*; 4 *Department of Chest Medicine, R.G. Kar Medical College and Hospital, Bagbazar TU, Kolkata, India*

**Keywords:** Directly observed treatment-short course, India, Kolkata, revised national tuberculosis control programme, tuberculosis

## Abstract

**Background:**

The Revised National Tuberculosis Control programme (RNTCP), India.

**Aim:**

To assess the impact of the expansion of the RNTCP in the case detection and treatment outcome.

**Materials and Methods:**

Reports of patients with tuberculosis (TB) diagnosed and treated under RNTCP from 2001 to 2005 under Bagbazar TB unit (TU), Kolkata, reviewed retrospectively.

**Results:**

Of 2814 cases registered between 2001 and 2005, 1268 were new smear-positive pulmonary TB (PTB), 308 were new smear-negative PTB and 536 were new extrapulmonary TB (EPTB). During that period, the new smear-positive case detection rate increased from 41 to 61 per lakh population, the annual total case detection rate increased from 87 to 142 per lakh and the treatment success rate reduced from 90% to 76%. The default and failure rates increased from 7% to 10% and from 3% to 10%, respectively.

**Conclusion:**

A steady increase was observed in the annual total case detection rate and annual new smear-positive case detection rate from 2001 to 2005, but the 3-month conversion rate and cure rate of new smear-positive patients were progressively decreased. Default rate and treatment failure rate of new smear-positive patients were also increased. So it needs extra attention and evaluation of this disappointing treatment outcome.

## INTRODUCTION

Tuberculosis (TB) remains a major public health problem in India. Every year approximately 18 lakh people develop TB and about 4 lakh die from this disease.[1] India accounts for one fifth of global incidence of TB and tops the list of 22 countries with high TB burden.[2] In India every day more than 40 thousand people become newly infected with the tubercle bacilli, more than 5000 develop TB disease and more than 1000 people die of TB.[3] The situation is worsened by low socioeconomic status and epidemic of human immunodeficiency virus (HIV) infection. Annual risk of TB infection (ARTI) is one of the important epidemiological indicators of the TB disease situation in a community. Currently, the average ARTI in India is 1.5%.[3] This means that there will be 75 new smear-positive cases, 75 new smear-negative cases, 38 re-treatment cases and 15 extrapulmonary cases, totaling to 203 cases per lakh population per year.[3] In India, the annual new smear-positive case detection rate was 66% in 2005 and treatment success rate was 86% in 2004.[4]

Despite the existence of a National Tuberculosis Control Programme since 1962, there was little effect on TB burden since 1992.[3] On recommendation of an expert committee a revised strategy to control TB was pilot-tested in 1993.[3] Full fledged Revised National Tuberculosis Control Programme (RNTCP) was started in 1997, and more than 97% of geographic coverage was achieved by the end of 2005.[4] The goal of RNTCP includes detection of at least 70% of new sputum-positive cases and achieving a cure rate of at least 85% among such cases in the population.[3]

World Health Organization (WHO) declared TB to be a global emergency in the year 1993.[5] The DOTS (Directly Observed Treatment-Short course) strategy is believed to be the most valuable strategy for TB control. At the end of 2003, a total of 182 countries in the world adopted and implemented DOTS.[6] Studies from resource-poor settings demonstrated that DOTS is an effective tool for TB control, and the World Bank report stated that TB chemotherapy is “one of the most cost effective of all health intervention”.[7]

Data (provided by Health and Family Welfare Department, Government of India) analysis of TB patients in India in the last 10 years demonstrated that the implementation of DOTS resulted in improved treatment success and a decrease in default rate. The present study aims to assess the impact of the implementation and expansion of DOTS on the trend of TB cases and their treatment outcome in the Bagbazar TU situated in the northern part of Kolkata city.

## MATERIALS AND METHODS

Bagbazar TU is located in the northern part of Kolkata city, with a population of about 4.96 million, of which 60% live in slum area. In this area, hygiene and education are very poor and accessibility to health services is limited. A pilot DOTS programme, supported financially and technically by RNTCP, was introduced in one region of Kolkata in 1994, gradually expanded to other areas including Bagbazar TU.

The RNTCP adopted the WHO recommendations and guidelines for treatment of TB patients and also for monitoring and evaluation of programme activities. Briefly, patient with signs and symptoms suggestive of TB were investigated and put on treatment. Patients with at least two sputum smears positive for Acid-Fast Bacillus, or one sputum-positive smear with either positive sputum culture or suggestive chest X-ray for TB were considered smear positive. Those with three negative smears were requested to undergo chest X-ray and treated with the antibiotics and then re-evaluated for diagnosis of smear-negative pulmonary TB (PTB). Diagnosis of extrapulmonary TB (EPTB) was made clinically and by the laboratory investigation.

After diagnosis of TB, patients were referred to the nearest DOTS center where they were registered for treatment. New smear-positive patients in the DOTS clinic received a six-month short-course therapy including thrice weekly supervised dose of rifampicin (R), isoniazid (I), pyrazinamide (Z), ethambutol (E) for two months, followed by self-administered RH for four months (2H_3_R_3_Z_3_E_3_/4H_3_R_3_). New smear-positive patients were monitored by smear examination at the end of the second and fourth month, and at the end of treatment. New smear-negative and EPTB cases received thrice weekly RHZ in the first two months followed by RH for four months (2R_3_H_3_Z_3_/4R_3_H_3_) and were monitored by regularity of attendance and clinical improvement. Sputum smear examination was done at the end of second month and at the end of treatment in smear-negative PTB. All forms of re-treatment patients received supervised thrice weekly dose of streptomycin, rifampicin, isoniazid, pyrazinamide, ethambutol for two months and one-month HRZE followed by RHE for five months (2R_3_H_3_Z_3_E_3_S_3_/1R_3_H_3_Z_3_E_3_/5R_3_H_3_E_3_).

The Bagbazar TB unit (TU) compiles information about all TB patients entered into the unit register and assigns a TB number to each patient in the unit TB register. The Bagbazar TU prepares report for case detection and treatment outcome quarterly and submits the report to the District Tuberculosis Officer, who is responsible for compiling the reports from all units, and submits them to the state TB officer. State TB Officer compiles reports from all districts and submits to the Central TB Division. All coordinators keep copies of the report for their documentation.

For this study, the data retained at the Bagbazar TB unit were collected for analysis. The case detection rate has been calculated by dividing the number of smear-positive cases by the WHO estimated number of smear-positive cases per 100,000 populations in the same year for the country. The Case Detection Rate and the proportion of smear-positive cases treated by Short Course Chemotherapy who successfully completed treatment; failure rate and the default rate have been considered as the main outcome variables. Ethical approval is not taken as the survey is based on retrospective data.

## RESULTS

Over the five-year period from 2001 to 2005, a total of 2814 patients with all forms of TB were registered for treatment under Bagbazar TU, which controls 7 DOTS centers. Of these 2814 patients, 1268 were new smear positive, 308 were new smear negative and 536 were new EPTB [Table 1]. There was a marked increase in the number of new PTB and new EPTB in the 5-year period. The case detection rate of all forms of TB increased from 87 per lakh in 2001 to 142 per lakh in 2005. The smear-positive case detection rate increased 1.5 times, from 41 per lakh (54%) in 2001 to 61 per lakh (81%) in 2005 compared with the national target of 70% [Table 1]. Estimated new smear-positive cases per lakh population based on ARTI data for the east zone of India is 75. The increase in the number of registered TB cases from 406 in 2001 to 703 in 2005 was probably due to the expansion and strict implementation of the DOTS strategy in the northern region of Kolkata. Though the population and absolute number of TB cases increased steadily from 2001 to 2005, the percentage of sputum smear-positive cases out of total new pulmonary cases on an average remains stabilized at 80%. It has been observed in this study that the number of smear-negative TB is less in this area. Percentage of new EPTB cases of all new cases increased slowly and steadily from 21% in 2001 to 29% in 2005 because more and more patients of TB with HIV had been reporting for treatment under DOTS although the number was still very low. Among patients who were tested for follow-up smear testing, the negative conversion rate at month 3 decreased from 88% in 2001 to 77% in 2005 [Table 1]. Cure rate for new sputum smear-positive patients decreased steadily from 90% in 2001 to 76% in 2005 [Table 2]. At the same time, the numbers of smear-positive re- treatment cases registered for treatment had increased from 98 in 2001 to 153 in 2005. This significant increase was probably due to more effective case detection, increasing number of patients attending the DOTS clinic and also due to failure of non-DOTS regimen before RNTCP. Percentage of re-treatment cases out of all smear-positive cases had not changed significantly from 2001 to 2005, indicates that on an average 33% of all sputum smear-positive patients belong to re-treatment category.

**Table 1:** Performance of revised national tuberclosis control programme case detection and smear conversion

Year	Population (in lakh)	Total patients initiated on treatment	Annual total case detection rate per lakh	New smear positive patients initiated on treatment	Annual new smear-positive case detection rate per lakh (%)	% of sputum positive of total new pulmonary cases	3-month conversion rate of new smear-positive patients (%)	Cure rate of new smear-positive patients (%)	No. of new smear-negative patients initiated on treatment	No. of new EP cases initiated on treatment	Percentage of new EP cases among all new cases	No. of smear-positive re-treatment cases initiated on treatment	Percentage of re-treatment cases of all smear-positive cases
2001	4.65	406	87	192	41 (54)	80	88	90	47	63	21	98	34
2002	4.72	411	87	189	40 (53)	78	78	82	54	64	21	94	33
2003	4.80	554	115	257	54 (72)	84	74	86	59	97	23	125	33
2004	4.88	740	152	329	67 (89)	82	82	81	71	159	28	165	33
2005	4.96	703	142	301	61 (81)	80	77	76	77	153	29	153	34
Total		2814		1268					308	536		635	

**Table 2:** Treatment outcome

Year	New smear positive						New smear negative						New extrapulmonary					
			
	Total no. of patients registered	Cure (%)	Completed	Died (%)	Failure (%)	Defaulted (%)	Total no. of patients registered	Cure	Completed (%)	Died (%)	Failure (%)	Defaulted (%)	Total no. of patients registered	Cure	Completed (%)	Died (%)	Failure (%)	Defaulted (%)
2001	192	90	0	0.5	2.8	6.7	47	0	93.6	2.1	2.1	2.1	63	0	93.6	0	0	6.3
2002	189	82	0	4.8	4.7	8.4	54	0	96.3	0	3.8	0	64	0	93.7	3.1	0	3.1
2003	257	86	0	2.3	4.6	6.6	59	0	89.8	0	0	10.2	97	0	94.8	2.1	0	3.1
2004	329	81	0	2.7	4.8	10.9	71	0	85.9	1.3	2.8	9.8	159	0	89.9	3.1	0	6.9
2005	301	76	0	3.3	9.6	9.9	77	0	83.1	2.6	1.3	11.7	153	0	91.5	3.9	0	4.5
Total	1268						308						536				

Of 1268 patients, new smear-positive patients registered from 2001 to 2005, 100% were treated with DOTS and evaluated for treatment outcome. The cure rate decreased from 90% in 2001 to 76% in 2005. The failure rate increased from 3% in 2001 to 10% in 2005 and default rate increased from 7% in 2001 to 10% in 2005 [Table 2]. In case of new smear-negative PTB, 94% patients completed treatment in 2001, whereas 83% completed in 2005. Treatment failure decreased from 2.1% to 1.3% and default increased from 2.1% to 11.6% in smear-negative PTB. With regard to EPTB, of 536 cases, 94% patients completed treatment in 2001, whereas 92% in 2005. Default rate decreased from 6% in 2001 to 5% in 2005 [Table 2].

## DISCUSSION

The DOTS strategy is a key factor to achieve success in TB control in India. The findings of this study indicate that, in line with the expansion and proper implementation of RNTCP, there is a steady increase in the annual total case detection rate and annual new smear-positive case detection rate. The RNTCP pilot project was launched in 1994 at Kolkata (Tangra) and Murshidabad in the state of West Bengal.[1] Under Bagbazar TU, 7 DOTS centers are functioning with maximum capacity. Stepwise scale-up was necessary in securing proper utilization of resources and to deal with challenges faced during implementation. For proper expansion and implementation of RNTCP, Bagbazar TU has involved several non-governmental organizations and private hospitals in the northern region of Kolkata.

Between 2001 and 2005, annual total case detection rate has increased 1.6 times. This may be due to increased awareness through Information Education Communication activities, better sputum microscopy and more number of EPTB cases reporting for treatment. More social awareness about AIDS and HIV has helped in more HIV-positive patients with TB to seek treatment under DOTS. The national target for sputum smear-positive case detection rate is 70%, which has already been achieved in 2003. This case detection rate is progressively increasing in the subsequent years. The most likely explanation for the increased number of reported cases may be due to the improved diagnostic facilities, including better sputum microscopy and increased detection of EPTB cases. Bagbazar TU includes one medical college (R.G. Kar Medical College), which receives numerous EPTB cases, which have been diagnosed with the facilities of a tertiary medical center. This increase may be partly contributed by increased HIV infection in some areas covered by Bagbazar TU, which is nearer to a large brothel. The number of sputum smear-negative TB patients initiated on treatment has increased progressively from 47 to 77 between the years 2001 and 2005.

In this study, the number of smear-negative TB has been observed to be less in this area. The probable explanation for this observation may be that there is a tertiary-level Medical College and Hospital catering to this area, as a result of which most cases are referred to this hospital for management. Further, as such, all the smear-negative patients attended to at this hospital are thoroughly evaluated and most of them turn out to be nontubercular cases, such as interstitial lung diseases, bronchiectasis and malignancy. Thus, after thorough evaluation of all smear-negative cases, only those found to be of tubercular etiology are referred to the RNTCP center for DOTS. Therefore, such thorough evaluation would not have been possible in the absence of tertiary-level referral Medical College and Hospital.

Second, a trend of injudicious prescription of antibiotics, especially Quinolones, by private practitioners has also been observed in this area. There is a possibility that a handful of TB patients who make their first visit to a private practitioner have an apparent suppression of symptoms and sense of well-being after receiving Quinolones for a long time. Subsequently, when symptoms reappear in these patients, they may turn out to be sputum-positive TB patients or some of them may move over to other areas in search of satisfactory treatment. Probably, this also contributes to the less number of smear-negative TB patients in this area.

Further, it should be noted that there are more sputum-positive patients registered for treatment than sputum-negative patients registered in many districts in different states of India.[8]

Negative smear conversion at the end of the third month (at the end of intensive phase, including delayed conversion) of DOTS treatment has declined from 88% to 77% between the year 2001 and 2005, which predicts that the treatment outcome has not reached the national average of 89%.[4] This may be explained by increasing number of drug-resistant TB. Perhaps, patients who were treated previously without any supervision before implementation of RNTCP under Government sectors and private practitioners may have formed the pool of suspected drug-resistant cases. Some patients may convert at 5 months, mainly seen in patients with high initial bacillary load. Because of the same reason the cure rate has declined from 90% in 2001 to 76% in 2005. Additional reason may be due to removal of large numbers of slums in the area, leading to migration of a large population to other area not covered by this TU; and thereby increasing the default rate. Numbers of re-treatment cases have increased steadily between 2001 and 2005 but the percentage of re-treatment cases among all smear-positive cases remain same throughout the last 5 years (33%).

The RNTCP status report shows favorable outcome throughout the country but unfortunately in the northern region of Kolkata, the cure rate is gradually declining from 90% in 2001 to 76% in 2005. Keeping similarity, the falling cure rate is accompanied by gradual increase in the failure rate and default rate in smear-positive PTB. Because of the increasing default rate and failure rate, cure rate is steadily decreasing. Approximately 33% of population of Kolkata lives in slum area. Bagbazar TU harbors many slums and migratory people from the different districts and adjoining states. Most of them are illiterate, poor laborers and daily wage earners. Few patients return to their native place after starting DOTS and few do not come to the DOTS center after becoming symptom-free. Our feedback and monitoring system may be not so developed, that we can trace out the missing patients; these patients have been included in defaulter category. Increased failure rate may be due to concomitant HIV infection and primary drug resistance. Because Bagbazar TU is nearer to a brothel, enzyme linked immunosorbent assay (ELISA) for HIV should be performed more intensively. But it is done in our country on voluntary basis. HIV testing may be done routinely rather than voluntarily in all patients with TB living in areas with a low literacy rate. By the time a patient with TB develops full-blown AIDS symptoms, they must have spread infection to other 10-15 people.[3]

Regarding smear-negative PTB and EPTB, percentage of patients completing treatment are though at par with the national average, but still they are also showing declining trends, which may come down to less than the national average in the near future. Here, the failure rate is not changed significantly in the 5 years probably because of the low bacillary load. But, default rate is increasing significantly in the sputum-negative PTB. The reason is probably the same as sputum-positive PTB cases and also due to increased number of alcoholics and drug addicts. When the entire country is showing favorable outcome of RNTCP, unfortunately one pocket of Kolkata is not showing encouraging outcome, which perhaps needs more attention regarding the factors responsible for it. To combat the problem of high default rate, evening DOTS center may be established in this area. The day-time workers who join their duties early in the morning and unable to attend the DOTS clinic during day time may be the ideal candidate for evening DOTS. Health education and stricter implementation of Information Education Communication activities in this area regarding free antitubercular drug supply, HIV prevention, etc. are needed. Problems such as social stigma of the disease, leading to providing false address, nonreporting to treatment center after symptomatic improvement, etc. should be dealt strictly. Study may be conducted to find the incidence of primary drug resistance, particularly in this pocket of the country. Other comorbid conditions, such as diabetes, should be checked in this area. Finally, monitoring and feedback system may be strengthened to find out the default cases early and native places of all patients should be recorded so that they can be traced to their native places also. For follow-up of the migrant patients, the electronic communication system between STOs of different states should be strengthened.
